# Supplementary material for: Robust Amino-Functionalized Mesoporous Silica Hollow Spheres Templated by CO2 Bubbles
Source: Molecules. 2021 Dec 22;27(1):53. doi: 10.3390/molecules27010053 (PMC8746618; doi:10.3390/molecules27010053)
Supplement: Supplementary file 1 [file molecules-27-00053-s001.zip › molecules-1490704-supplementary.pdf]

molecules

Supplementary Materials

Robust Amino-Functionalized Mesoporous Silica Hollow Spheres Templated by CO_2 Bubbles

Hongjuan Wang [1,2,†], Xuefei Liu [1,†], Olena Saliy [2,3], Wei Hu [1,2,*] and Jingui Wang [1,*]

[1] School of Chemistry and Chemical Engineering, Qilu University of Technology (Shandong Academy of Sciences), Jinan 250353, China; hongjuanwang2015@163.com (H.W.); paowuxian124@163.com (X.L.)
[2] Kyiv College, Qilu University of Technology (Shandong Academy of Sciences), Jinan 250353, China; saliy.oo@knutd.edu.ua
[3] Department of Industrial Pharmacy, Faculty of Chemical and Biopharmaceutical Technologies, Kyiv National University of Technologies and Design, 01011 Kyiv, Ukraine
* Correspondence: weihu@qlu.edu.cn (W.H.); JGWang@qlu.edu.cn (J.W.)
† These authors contributed equally to this work.

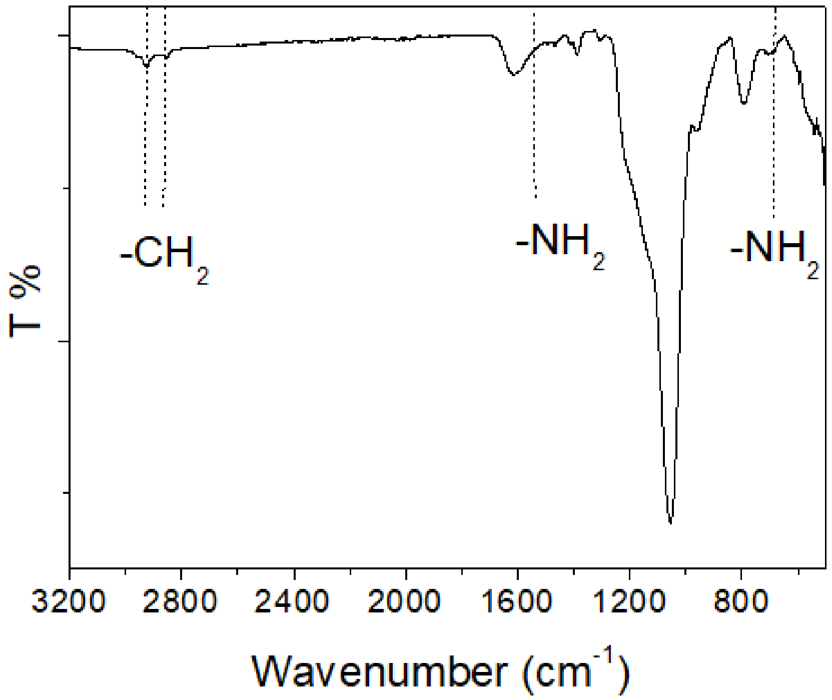

Figure S1. FTIR spectra of amino-functionalized mesoporous silica hollow spheres templated by CO_2 bubbles. The absorption bands at about 1550 cm^{-1} and 685 cm^{-1} were corresponding to the vibration of amine. The adsorption bands at 2930 cm^{-1} and 2858 cm^{-1} indicated the C–H stretching vibration.

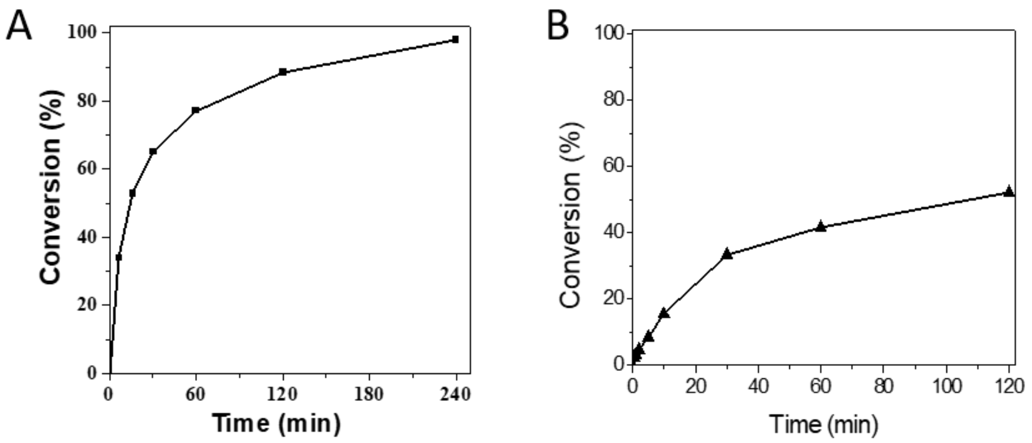

Figure S2. Knoevenagel reaction curve of (**a**) our sample of amino-functionalized mesoporous silica hollow spheres and (**b**) control sample of traditional amino-functionalized MCM-41 mesoporous silica.

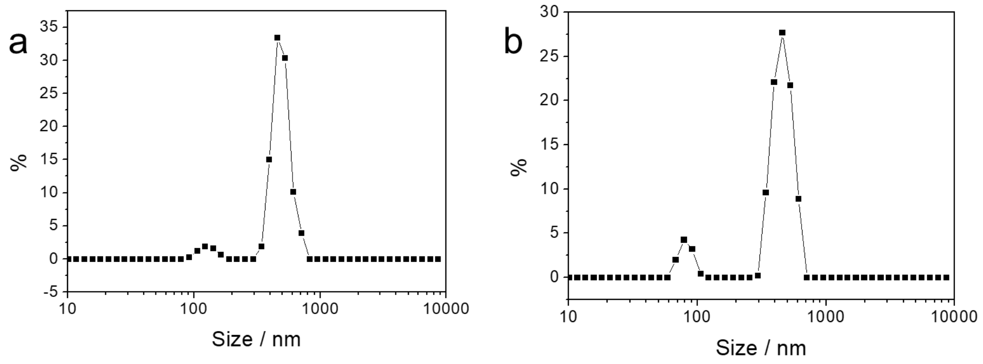

Figure S3. Particle size distribution measured by DLS (**a**) before and (**b**) after adding metoprolol to the spheres.

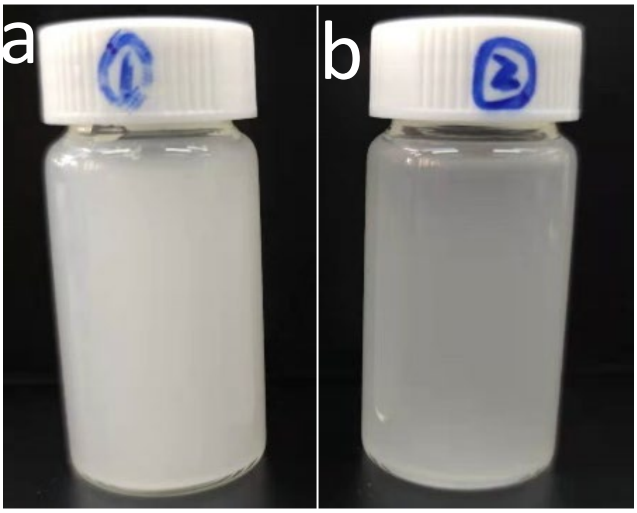

Figure S4. Photos of synthesized hollow spheres dispersed in (**a**) DI water and (**b**) ethanol.
